# Infectious native valve endocarditis by *Streptococcus agalactiae* species: Case report of pathogen identification only through metagenomic sequencing technology

**DOI:** 10.1097/MD.0000000000029360

**Published:** 2022-07-08

**Authors:** Ruoxin Wang, Xuejie Cao, Fang Wu, Jinlong Zhao, Liang Fu, Ziming Yuan, Yinkai Ni, Zonghui Chen, Feng Li

**Affiliations:** a Department of Cardiovascular Surgery, Shanghai Jiaotong University Affiliated Sixth People’s Hospital, Shanghai, China; b Genoxor Medical Science and Technology Inc., Shanghai, China; c Department of Intensive Care Unit, Shanghai Jiaotong University Affiliated Sixth People’s Hospital, Shanghai, China.

**Keywords:** infective endocarditis, mNGS, *Streptococcus agalactiae*

## Abstract

**Introduction:**

*Streptococcus agalactiae* is a common pathogen in infective endocarditis, but the positive rate of traditional blood culture diagnosis is not high. It is challenging to obtain a good outcome in the absence of pathogen information for patients with infectious endocarditis.

**Patient concerns and diagnosis:**

Here, we report the case of a patient with infective endocarditis caused by *S. agalactiae*. The initial manifestations of this patient were coma, urinary incontinence, and fecal incontinence and had no history of heart disease or infectious diseases before admission.

**Interventions and outcomes:**

When the blood culture was negative 3 consecutive times, the pathogen *S. agalactiae* was diagnosed in a timely and accurate manner by metagenome sequencing. Eventually, the patient was discharged following surgery and antibiotic treatment.

**Conclusions:**

For IE patients with infective endocarditis, metagenome sequencing is a valuable and selective tool for rapid, sensitive, and accurate pathogen detection, especially when the blood culture is negative.

## 1. Introduction

*Streptococcus agalactiae*, also known as group B Streptococcus (GBS), is a serious pathogen that causes severe anthropozoonosis in a broad range of hosts, from aquatic animals to mammals, including humans.^[1]^ *S. agalactiae* is also a common pathogen in infective endocarditis (IE), but the positive rate of traditional blood culture diagnosis is not high.^[2,3]^ Although IE caused by this microorganism is rarely diagnosed by blood culture, most cases require early treatment plans because of serious complications such as major embolism or heart failure with rapid valve destruction.^[4]^

Blood culture is the gold standard for detecting pathogens in infective patients. However, many factors affect the positive rate of blood cultures. The culture-low positive rate may be attributed to the use of empirical antibiotics. In addition, harsh culture conditions and slow reproduction of pathogens (HACEK group, *Bartonella*, *Brucella*, *Chlamydiophila*, and *Mycobacterium*) also cause a high false-negative rate.^[5,6]^ Unlike traditional blood culture testing, the advent of metagenomic next generation sequencing (mNGS) technology coupled with ongoing improvements in computing power has enabled clinicians to rapidly detect pathogens. Here, we report the case of a patient with IE caused by *S. agalactiae*. The patient underwent mNGS testing and this pathogen was confirmed before surgery, but blood culture was continuously negative during the perioperative period. Consequently, the patient underwent surgery and targeted antibiotic therapy, and recovered well.

### 1.1. Case presentation

**Figure 1.**
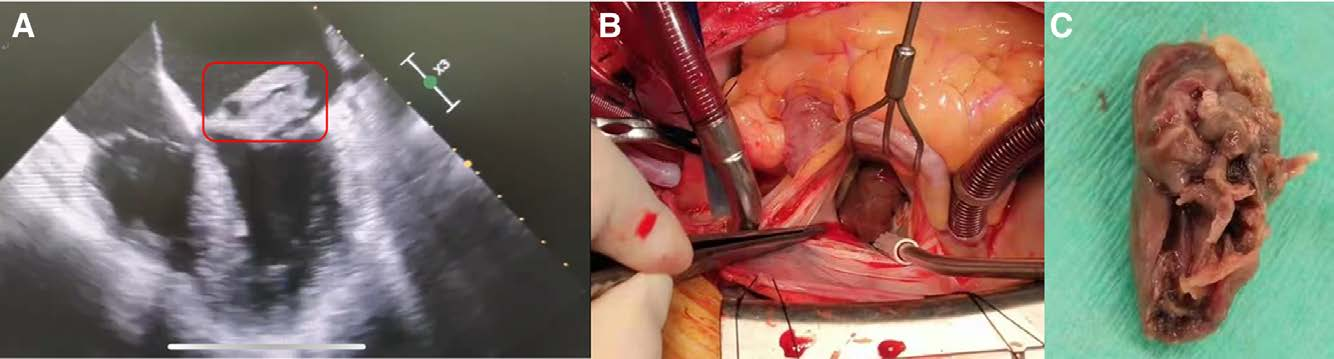
Clinical information of infective endocarditis. (A) Transesophageal echocardiography showing a 22 × 15 mm ectogenic and hypermotile mass on the left atrial mitral valve leaflets. (B) An intraoperative picture of mitral biological valve replacement under general anesthesia. (C) Vegetation in mitral valve.

**Figure 2.**
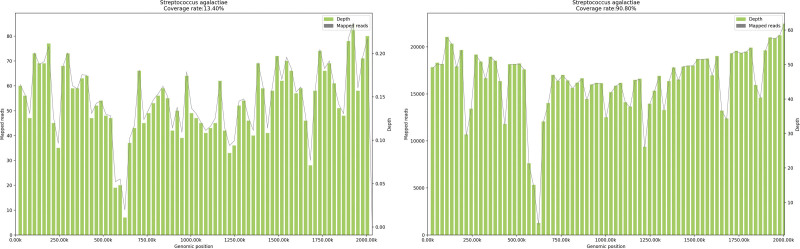
Molecular detection technology-mNGS. Genome coverage of *Streptococcus agalactiae*. (Left) Genome coverage map of Blood-1; (Right) Genome coverage map of the vegetation. mNGS = metagenomic next generation sequencing.

A 59-year-old woman was admitted to the emergency room with coma along with urinary and fecal incontinence. The patient’s medical history included no definite comorbidities or infectious diseases, including the respiratory or urogenital tract, before admission. The following physical findings were observed during her visit: body temperature, 38.0°C; blood pressure, 94/58 mm Hg; pulse rate, 90/min; and a mild diastolic heart murmur at the apex on auscultation. Laboratory findings revealed a high white blood cell count (18.2 × 10^9^/L) and CRP (135.65 mg/L) and C-reactive value (11.3 × 10^9^/L). Blood analysis shows that the lower PH (6.91) and higher PaO_2_ (135 mm Hg). Cardiac ultrasonography showed isoechoic masses (24 × 17 mm) on the mitral leaflets in the left atrium. Horizontal atrioventricular shunts were not observed. The left atrial anterior-posterior diameter was 34 mm, the left ventricular anterior-posterior diameter (diastolic) was 39 mm, the left ventricular anterior-posterior diameter (systolic) was 28 mm, and the ejection fraction was 54%. The brain MRI showed multiple cerebral infarctions in the right thalamus, cerebellar hemisphere, bilateral frontal parietal lobe, and left occipital lobe (subacute stage). Blood culture tests were conducted in the next 3 continuers. We suspected IE and started using an empirical antibiotic (IV vancomycin 20 mg/kg loading dose) and then 15 mg/kg every 12 hours. We referred this patient to the cardiovascular surgery department the next day. Three days after vancomycin administration, the patient still complained of dyspnea. Moreover, continuous norepinephrine infusion is required for hypotension. Three sets of blood culture test results were negative. A clinical routine test was performed again, which showed a WBC 32.1 × 10^9^/L, absolute and relative values of neutrophil cells (28.1 × 10^9^/L, 87.5%). Cardiac ultrasound showed isoechoic masses (23 × 16 mm) on the mitral leaflets in the left atrium, perforation of the anterior mitral leaflets with moderate mitral regurgitation, left atrial anterior-posterior diameter of 40 mm, left ventricular anterior-posterior diameter (diastolic) of 42 mm, left ventricular anterior-posterior diameter (systolic) of 28 mm, and an ejection fraction of 63%. Due to highly suspected IE, even negative blood culture results were obtained and the modified Duke criteria were not met. The blood of our patient was drawn and sequenced for mNGS. Encouragingly, the results for *S. agalactia* were reported on the same day (Fig. 2, left), and mitral biological valve replacement was performed under general anesthesia the next day. Transesophageal echocardiography and vegetation in mitral valve were shown in Figure 1. Mitral valve vegetation was sent for culture and mNGS, which detected *S. agalactiae* (Fig. 2, right) and confirmed the diagnosis of IE. After 4 weeks of vancomycin treatment, the patient’s inflammatory indices and vital signs were normal. To confirm that the bacteremia was fully cured, we performed blood culture and mNGS, which yielded negative results. The patient was discharge on postoperative day 31. The specific treatments and blood examinations of the patients are shown in Table 1.

**Table 1:** Blood leukocyte counts as well as blood neutrophil and monocyte in the the 59-year-old woman.

Admission time	WBC (10^9^/L)	Neutrophils (10^9^/L)	Monocyte (10^9^/L)	Cardiac ultrasound	Other
DAY 1	18.2	11.3	4.4	Supravalvular isoechoic mass of mitral valve in left atrium (24 × 17 mm, Atrioventricular horizontal shunt was not observed, Left atrium anterior-posterior diameter 34 mm, Left ventricular anteroposterior diameter in Diastolic period and Systolic period are 39 mm and 28 mm, Ejection fraction 54%	-
DAY 2				Refer to cardiovascular surgery	-
DAY 5	32.1	28.1	1.5	Supravalvular isoechoic mass of mitral valve in left atrium (23 × 16 mm, Anterior mitral perforation with moderate mitral regurgitation, Left atrium anterior-posterior diameter 40 mm, Left ventricular anteroposterior diameter in Diastolic period and Systolic period are 42 mm and 28 mm, Ejection fraction 63%	Vancomycin treatment
DAY 5				Identify pathogens through mNGS from blood	
D-DAY 1				Biological valve replacement of mitral valve under general anesthesia	
D-DAY 2	20.8	18.3	0.5	-	
D-DAY 4				Pathogen detected in vegetation through mNGS	
D-DAY 8	7.3	5.5	0.2	Left atrium anterior-posterior diameter 32 mm, Left atrium anterior-posterior diameter 40 mm, Left ventricular anteroposterior diameter in Diastolic period and Systolic period are 42 and 30 mm, Ejection fraction 53%	
D-DAY 30	7.7	5.5	0.8	Left atrium anterior-posterior diameter 34 mm, Left ventricular anteroposterior diameter in Diastolic period and Systolic period are 44 and 28 mm, Ejection fraction 67%	
D-DAY 31				Blood culture and mNGS test negative for pathogens, Stop antibiotic treatment	

Initiation of *Streptococcus agalactis*-identified and specific therapy is marked by words.

mNGS = metagenomic next generation sequencing.

## 2. Discussion

IE refers to inflammation caused by infection of the endocardium or heart valves by microorganisms, such as bacteria, fungi, and viruses. Despite advances in medical intervention, IE remains a disease that can lead to morbidity and mortality.^[7]^ According to reports, the incidence of IE is approximately 3 to 10 cases per year for every 100,000 patients.^[8]^ For IE, the treatment measures are mainly aimed at the 2 links between bacteremia and the heart. Therefore, surgery and antibiotic treatment are currently the treatment options for patients with IE.^[9,10]^ For patients undergoing surgery, antibiotic prophylaxis of infection should also be administered after surgery. In summary, for IE patients, rapid and accurate identification of the pathogen is one of the most important clinical tasks.

The incidence of *S. agalactiae* infection has been increasing in both the elderly and those with comorbid conditions. *S. agalactiae* IE often develops in patients with underlying diseases such as diabetes mellitus, liver cirrhosis, malignancy, and abnormalities in immune responses.^[11]^ In our case, the patient has always been in good health and has no definite cardiac problems. Nevertheless, the patient was diagnosed with severe endocarditis caused by *S. agalactiae*. and is progressing rapidly. However, IE of the left heart caused by *S. agalactiae* infection is not accompanied by obvious skin lesions; therefore, it is easy to miss the diagnosis in the early stages.^[12]^ Blood cultures of *S. agalactiae* are also easily affected by antibiotics.^[13]^ If the pathogen cannot be identified by traditional detection methods, more sensitive detection methods are required.

**Figure 3.**
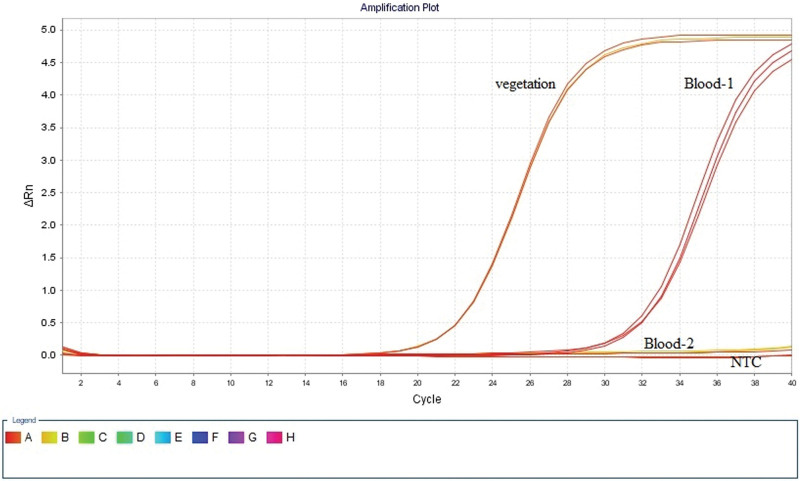
Molecular detection technology-qPCR. Real-time qPCR amplification curve confirms *Streptococcus agalactiae*.

The pathogen responsible endocarditis is mainly a single strain with low complexity, and disease progression is very fast. Therefore, antibiotics selected based on antibiotic susceptibility test will be more suitable for patients with IE. mNGS detects all microbial nucleic acids in a sample and identifies the suspected pathogenic microorganisms. The operation cycle issued by the results received from the samples can be controlled within 24 hours, and tens of thousands of microorganisms and drug resistance genes can be covered at one time. Overall, according to our experience, mNGS is a valuable tool for rapid, sensitive, and accurate pathogen detection (the bacteria detected were validated by PCR) (Fig. 3). Our report illustrates the importance of mNGS as a detection tool for IE pathogens, especially when the blood culture is negative. It also indirectly proved that culture, together with mNGS of blood and valve samples, may enhance diagnostic yield.

## Author contributions

Conceptualization: R.W., F.L.

Data curation: F.W., J.Z., Z.C.

Validation: L.F., Z.Y., Y.N.

Writing – original draft: R.W., X.C.

Writing – review & editing: F.L.
